# Time intervals in the pathway to diagnosis and treatment of patients with breast and gynecological cancer

**DOI:** 10.3389/fmed.2025.1655888

**Published:** 2025-10-17

**Authors:** Blanca Sánchez Galindo, Joseba Rabanales Sotos, Ángel López González, Marta Castaño Díaz, Carmen María Sánchez Martínez, Jesús López-Torres Hidalgo

**Affiliations:** ^1^Albacete Integrated Care Management, Health Service of Castilla-La Mancha, Albacete, Spain; ^2^Albacete Nursing School, University of Castilla-La Mancha, Albacete, Spain; ^3^Valencia Health System, Valencia, Spain; ^4^Albacete Medicine School, University of Castilla-La Mancha, Albacete, Spain

**Keywords:** breast neoplasms, delayed diagnosis, early detection of cancer, female genital neoplasms, primary health care, public health

## Abstract

**Background:**

Breast and gynecological cancer have a high prevalence and a significant impact on public health. It is important to note that the time intervals until diagnosis and treatment influence the prognosis. The objective was to describe the delay in the diagnosis of breast and gynecological cancer and to identify the variables related to the patient, healthcare and the disease that intervene in the time interval until diagnosis and treatment.

**Methods:**

We conducted a retrospective study (2014–2023) following a cohort of women with breast and gynecological cancer, from the onset of symptoms to the start of treatment. The study included 722 women from 30 general practice clinics in Albacete, Spain, and data were obtained from both primary care and hospital settings.

**Results:**

Among breast cancer patients, 150 (25.7%) had been diagnosed through screening, and among those diagnosed with cervical cancer, 14 (37.8%), it was not possible to calculate some time intervals. In breast cancer the variables associated with a total time interval (from first symptoms to start of treatment) of more than 90 days were: age over 50 and symptoms other than a breast lump. In gynecological cancer, the related variables were: no family history and having attended the health center for the first consultation. In the diagnostic interval (from first consultation to diagnosis), the variables associated with a duration of more than 30 days were: presenting with fewer than two risk factors in breast cancer and first consultation at the health center in gynecological cancer.

**Conclusion:**

Most patients with breast and/or gynecological cancer are diagnosed in the early stages of the disease, except in the case of ovarian cancer. Most breast and cervical tumors are not diagnosed through screening. The time interval that most influences the total interval is the diagnostic interval, which includes the primary care interval. The treatment interval is high in most tumors, exceeding the recommended time. The results provide useful information for proposing improvements in access to diagnostic and therapeutic resources, as well as preferential referral circuits to improve early detection and prognosis of the disease.

## Introduction

The general practitioner’s (GP) practice is the principal setting where cancer is first suspected and where it becomes necessary to assess whether the patient’s symptoms pose a high likelihood of suffering from cancer (1).

Breast cancer is the most frequent tumor among women and accounts for 23% of all new diagnoses of cancer (2). Furthermore, the incidence of gynecological cancers has been rising due to inappropriate lifestyle patterns, dietary habits and genetic factors. Hence, endometrial, ovarian and cervical cancers represent more than one third of all cancers in women worldwide, reaching an incidence of 30.3 cases per 100,000 population per year (3).

Breast and gynecological cancers sometimes share common risk factors, such as age of onset, age at menarche or menopause, absence of breastfeeding, age at first pregnancy, alcohol and tobacco consumption, and BRCA mutations (4). These are important risk factors in addressing women’s health and especially in addressing cancer, both breast and gynecological, allowing for a common perspective when conducting epidemiological studies on cancer in women or when proposing public health measures aimed at improving the overall health of the female population.

Early diagnosis of breast cancer and various gynecological cancers is essential to reduce morbidity and mortality, which can be achieved through screening and early identification of signs and symptoms. Breast and cervical cancer screening programs facilitate the detection of these cancers in asymptomatic patients. Screening healthy women for cervical cancer using cervical cytology has reduced the incidence and mortality of this type of cancer by 70–80% (5). Breast cancer screening using mammography reduces breast cancer mortality by 20% and prevents one death from breast cancer for every 235 women screened over 20 years (6).

Cancers are often symptomatic and these symptoms contribute to a rapid diagnosis, which makes it possible to treat cancer successfully. According to the guidelines issued by the National Institute for Health and Care Excellence (NICE Guidelines), the presence of a symptom leading to diagnosis of cancer with a positive predictive value (PPV) of 5% or more would justify initiating the diagnostic study. The signs and symptoms with the highest PPV for breast and gynecological cancer include breast nodule, abnormal vaginal bleeding, postmenopausal metrorrhagia, and bloating and/or pain, among others (4, 7, 8).

Recognition of suspected cancer symptoms, both by healthcare professionals and patients, leads to early diagnosis and significantly increases the chances of receiving effective treatment (9). Furthermore, early diagnosis is considered more cost-effective than diagnosis at later stages due to the high costs of advanced-stage therapies (8). With the exception of breast and cervical cancers, there are currently no screening tests available for other gynecological cancers, meaning that their detection depends on the recognition of symptoms by women and their family doctors (10).

The probability of a longer time interval until diagnosis is higher in patients with non-specific symptoms, such as abdominal distension in ovarian cancer, than in those with typical symptoms, such as postmenopausal bleeding in endometrial cancer (11). Longer time intervals are associated with decreased survival (12, 13), as the “diagnostic interval” (DI) influences the type of treatment the patient will receive (14).

The Aarhus Statement (15) lays down a series of definitions of the various time intervals between symptom onset and treatment initiation: the “Patient interval” is defined as the time elapsed between detection of first symptoms and initial consultation, and is influenced by prior knowledge of cancer warning symptoms. In primary care, the time interval between the first consultation and the referral of the patient to hospital or the request for tests is particularly important, but the duration of the “interval attributable to the healthcare system” (HCI), or the time from the request for tests and/or referral to the start of treatment, is conditioned by waiting lists and access to diagnostic tests from primary care.

In general, despite the fact that a proportion of cancers are diagnosed at the presymptomatic stage, most patients already present with symptoms at the time of diagnosis. That said, however, there is an inversely proportional relationship between survival and the time that elapses until diagnosis and treatment of the disease (4).

The main aim of this study was thus to describe diagnostic delay in breast and gynecological cancer in primary care, and identify the variables of the patient (age, family history of breast or gynecological cancer, and comorbidity), healthcare process (participation in breast or cervical cancer screening, place of initial consultation and priority of referral) and disease (stage, symptoms of presentation and risk factors), which intervene in the time interval until diagnosis and treatment.

## Materials and methods

We carried out a retrospective observational study on a cohort of women aged 18 years and over, diagnosed with breast and/or gynecological cancer across the period 1/1/2014–31/12/2023, with data being obtained from both primary and hospital care settings from onset of first symptoms of the disease until treatment initiation. The participants were drawn from 30 family medicine practices at health centers serving Health Areas IV, VB and VIII of the city of Albacete, situated in the Castile-la Mancha Autonomous Region in the south-east of Spain. The study was approved by the Clinical Research Ethics Committee of the Albacete University Teaching Hospital Complex.

Based on morbidity lists furnished by the Turriano Information System (computerized primary-care clinical histories kept by the Castile-La Mancha Health Service), all women diagnosed with breast and/or gynecological cancer during the study period were consecutively selected (inclusion criterion). Patients were identified using the International Classification of Diseases, 9th Revision (ICD-9) and/or International Classification of Primary Care, 2nd edition (ICPC-2) codes, with the following exclusion criterion being applied: any tumor not classifiable as a malignant neoplasm and women whose medical records contained insufficient information to identify most of the time intervals until diagnosis and treatment.

**Figure 1:**
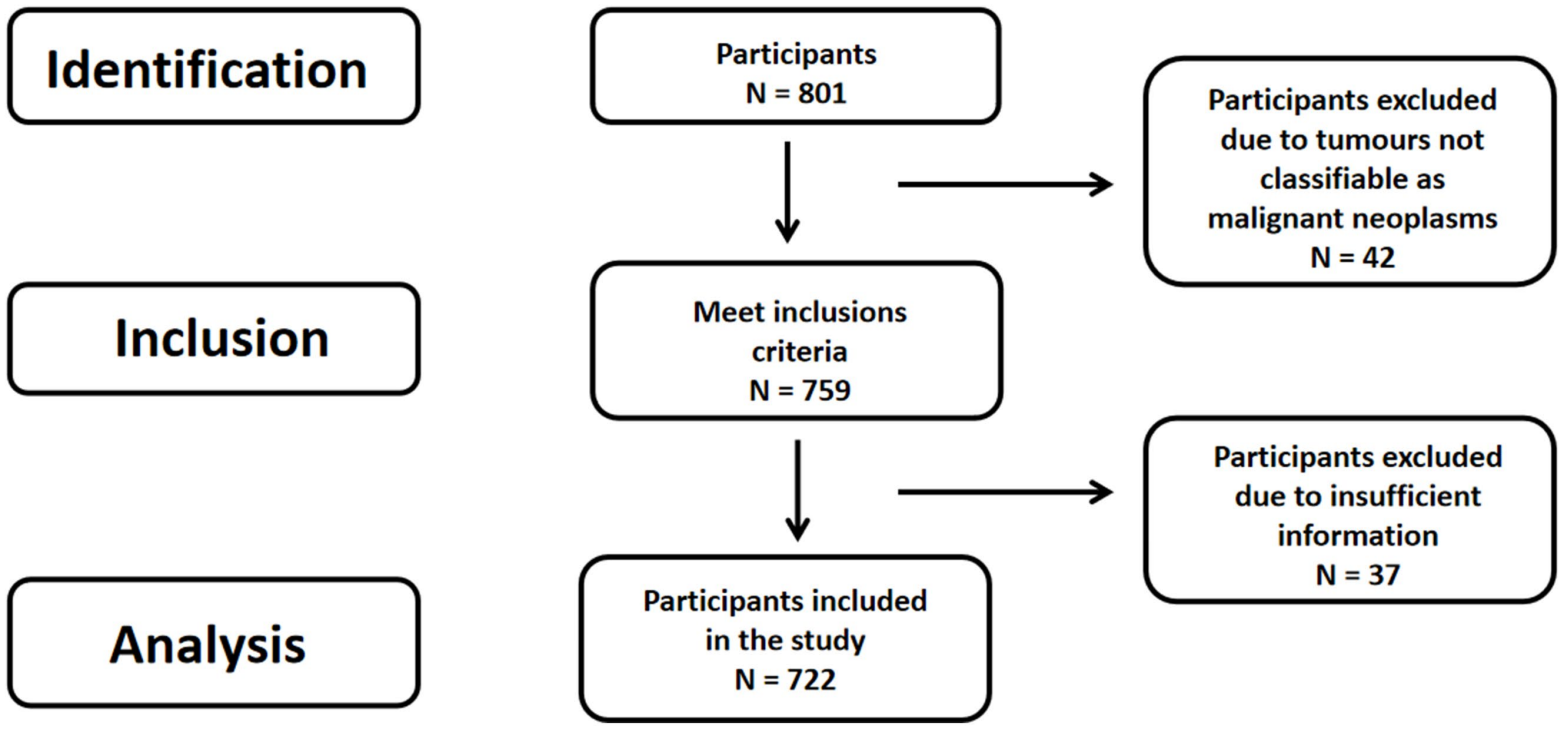
Flowchart of participating population.

A total of 722 patients were diagnosed with breast or gynecological cancer and included in the study (Figure 1), and yielded the following case breakdown: 584 with breast cancer; 52 with endometrial cancer; 37 with cervical cancer; 43 with ovarian cancer; and 6 with vulvar cancer. Although statistical power is very limited in cases of gynecological cancer, in those of breast cancer we check retrospectively that sample size allows for groups of patients to be compared with a 95% confidence level, a statistical power of 90%, and a standardized difference of means of 0.2–0.3 in the case of unknown variances. This sample size also allows, in the case of breast cancer, for estimates to be made with a confidence level of 95% and a minimum precision of ± 4%, which drops to 14–16% in cases of endometrial, cervical and ovarian cancer. The raw data is a Supplementary file that is part of the article.

The study variables included the following characteristics; patient (age, family history of breast cancer in first-degree blood relatives, and comorbidity); healthcare process (participation in screening, place of initial consultation, and priority of referral); and disease (tumor stage, symptoms of presentation and risk factors, including alcohol consumption and tobacco use, obesity, nulliparity, age of menarche, age of first gestation, age of menopause, and exposure to hormone therapy). The time intervals considered (15) were: “Patient interval” (PI) (time elapsed between detection of first symptoms and initial consultation); “Primary care interval” (PCI) (time elapsed between initial consultation and referral of patient to hospital, which includes the “Physician interval” (PHI) or time elapsed between initial consultation and first test requested in primary care, in those cases where this has been requested prior to referral); “Healthcare interval” (HCI) (time elapsed between first test requested by the GP and treatment initiation, which includes the “hospital care interval” (HOSPI) or time elapsed between referral and treatment initiation); “Diagnostic interval” (DI) (time elapsed between initial consultation and diagnostic confirmation); “Treatment interval” (TTI) (time elapsed between diagnostic confirmation and treatment initiation); and “Total interval” (TI) (time elapsed between symptom onset and treatment initiation).

All the data obtained were included in a study-specific electronic case-report form and then analyzed using the IBM SPSS statistics program version 28.0. The analysis strategy consisted of a description of the study variables, and for each type of cancer, a comparative analysis was performed of the different time intervals between women with different characteristics, using tests to compare means in independent groups (Student’s *t*-test) when the normality of the data was verified using the Kolmogorov–Smirnov test. For variables without a normal distribution or in comparison groups with fewer than 30 cases, a non-parametric test (Mann–Whitney U, using a significance level of *p* < 0.05) was used. Using multivariate analysis models (logistic regression), we ascertained which variables were associated with a “TI” length of more than 90 days and a “DI” length of more than 30 days in both breast and gynecological cancer, avoiding possible confounding factors and eliminating from both models any variables that failed to bring about an important change in the odds ratio (OR). It was decided to evaluate gynecological cancers jointly because they are tumors that develop in the structures of the female reproductive system and often share both the presenting symptoms and the usual diagnostic methods. The study variables were included in both models using the forward stepwise method, remaining in them when they produced a significant change in the value of the coefficients (the entry and exit criteria were *p*-value 0.05 and 0.10, respectively). In all models, the number of events per independent variable was greater than 10. Among the candidate variables (age, first-degree family history of breast and/or gynecological cancer, comorbidity, place of first consultation, referral priority, breast or cervical cancer screening follow-up, stage, number of risk factors and presenting symptoms), those that had previously shown a statistically significant association were included, and a complete case analysis was used in all cases. The fit was checked using the Hosmer-Lemeshow test, checking for *p*-values greater than 0.05.

## Results

**Figure 2:**
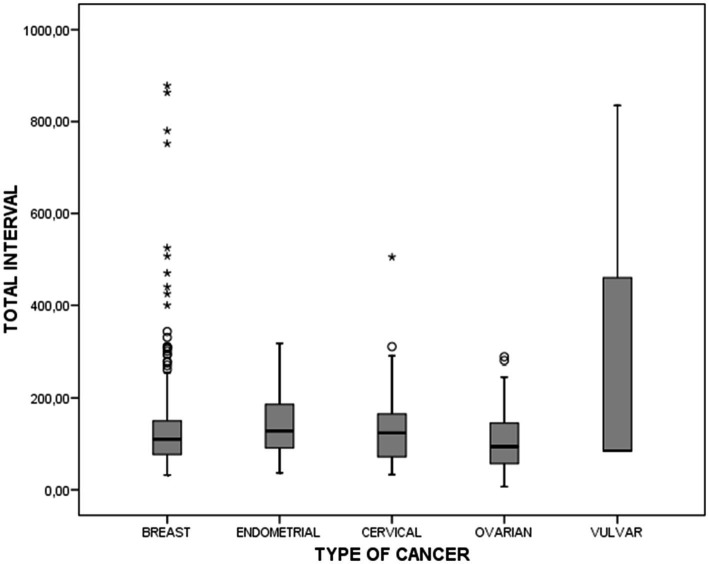
Box plot representation of the total interval in each type of cancer.

The patient flow diagram is shown in Figure 1, and the characteristics of the 722 participants studied are described in Table 1. The description of the time intervals considered in the study of diagnostic delay is shown in Table 2 and Figure 2. A sensitivity analysis was performed excluding values above the 99th percentile of each time interval, and it was found that the main findings were not altered. In the case of breast cancer patients, 150 (25.7%) had been diagnosed through a screening program and 14 (37.8%) of those diagnosed with cervical cancer. In these cases, it was not possible to calculate the patient, primary care, doctor, healthcare system and total intervals. The highest mean values for the “PCI” and “PHI” corresponded to ovarian cancer, with 30.8 and 11.6 days, respectively. On the other hand, the “HCI” and “HOSPI” had the highest mean values for cervical cancer (116.3 and 102.4 days, respectively).

**Table 1:** Characteristics of participants and the healthcare process.

Characteristics	Breast (*n* = 584)	Endometrial (*n* = 52)	Cervical (*n* = 37)	Ovarian (*n* = 43)	Vulvar (*n* = 6)
Age (years)					
Under 50	223 (38.2%)	4 (7.7%)	17 (45.9%)	11 (25.6%)	0 (0%)
50 to 64	187 (32.0%)	22 (42.3%)	11 (29.7%)	18 (41.9%)	4 (66.7%)
65 or over	174 (29.8%)	26 (50.0%)	9 (24.3%)	14 (32.6%)	2 (33.3%)
Tumor stage					
Stage I	214 (36.6%)	34 (65.4%)	16 (43.2%)	1 (2.3%)	4 (66.7%)
Stage II	251 (43.0%)	8 (15.4%)	8 (21.6%)	4 (9.3%)	0 (0%)
Stage III	93 (15.9%)	10 (19.2%)	11 (29.7%)	15 (34.9%)	2 (33.3%)
Stage IV	26 (4.5%)	0 (0%)	2 (5.4%)	23 (53.5%)	0 (0%)
Family history of breast and/or gynecological cancer					
Yes	112 (19.2%)	3 (5.8%)	3 (8.1%)	5 (11.6%)	0 (0%)
No	472 (80.8%)	49 (94.2%)	34 (91.9%)	38 (88.4%)	6 (100%)
Symptoms and signs of presentation					
Breast lump	378 (66.3%)	-	-	-	-
Nipple discharge	19 (3.3%)	-	-	-	-
Mastodynia	24 (4.1%)	-	-	-	-
Nipple inversion	47 (8.0%)	-	-	-	-
Other	4 (0.7%)	3 (5.7%)	8 (21.6%)	3 (6.9%)	2 (16.7%)
Abdominal pain	-	-	-	22 (51.2%)	-
Bloating	-	-	-	25 (58.1%)	-
Postmenopausal metrorrhagia	-	45 (86.5%)	12 (32.4%)	3 (7.0%)	-
Intermittent bleeding	-	5 (9.6%)	4 (10.8%)	5 (11.6%)	-
Vulvar ulcer	-	-	-	-	5 (83.3%)
Weight loss	-	-	-	4 (9.3%)	-
Place of initial consultation					
Health center	426 (72.9%)	42 (80.8%)	17 (45.9%)	24 (55.8%)	4 (66.7%)
Hospital emergency service	3 (0.5%)	10 (19.2%)	6 (16.2%)	19 (44.2%)	2 (33.3%)
Other	5 (0.9%)	0 (0%)	0 (0%)	0 (0%)	0 (0%)
Screening	150 (25.7%)	0 (0%)	14 (37.8%)	0 (0%)	0 (0%)
Other health problems					
None	229 (39.2%)	11 (21.2%)	20 (54.1%)	17 (39.5%)	3 (50.0%)
1 or 2	287 (49.1%)	27 (51.9%)	15 (40.5%)	22 (51.2%)	3 (50.0%)
3 or more	68 (11.6%)	14 (26.9%)	2 (5.4%)	4 (9.3%)	0 (0%)
Risk factors					
None	114 (19.5%)	3 (5.8%)	2 (5.4%)	5 (11.6%)	1 (16.7%)
1 or 2	353 (60.4%)	33 (63.5%)	20 (54.1%)	29 (67.4%)	5 (83.3%)
3 or more	117 (20.0%)	16 (30.8%)	15 (40.5%)	9 (20.9%)	0 (0%)

*n*, number of cases; %, percentage; “-”, insufficient data for these variables.

**Table 2:** Time intervals in diagnosis of breast and gynecological cancer.

Time intervals (days)	Breast (*n* = 584) Median IQR (P25 - P75)	Endometrial (*n* = 52) Median IQR (P25 - P75)	Cervical (*n* = 37) Median IQR (P25 - P75)	Ovarian (*n* = 43) Median IQR (P25 - P75)	Vulvar (*n* = 6) Median IQR (P25 - P75)
“Patient interval”* Time elapsed between first symptoms and initial consultation.	15 23 (7–30)	15 29 (5–34)	22 46 (8–54)	15 31 (7–38)	10 725 (5–730)
“Primary care interval”** Time elapsed between initial consultation and referral to hospital	14 26 (6–32)	0 0 (0–0)	0 0 (0–0)	0 34 (0–34)	17 58 (4–62)
“Physician interval”* Time elapsed between initial consultation and first test requested in primary care	0 0 (0–0)	0 0 (0–0)	0 0 (0–0)	0 13 (0–13)	- - -
“Healthcare interval”* Time elapsed between first test requested by the physician and treatment initiation	85 55 (61–116)	106 60 (79–139)	93 80 (54–134)	54 98 (30–128)	80 30 (75–105)
“Hospital care interval” Time elapsed between patient referral and treatment initiation	69 41 (48–89)	122 59 (83–142)	85 75 (59–134)	53 78 (32–110)	60 48 (37–85)
“Diagnostic interval” Time elapsed between initial consultation and diagnostic confirmation	35 31 (21–52)	42 61 (26–87)	45 41 (22–63)	55 75 (30–105)	37 52 (16–68)
“Treatment interval” Time elapsed between diagnostic confirmation and treatment initiation	40 36 (24–60)	49 48 (27–75)	45 37 (34–71)	5 27 (0–27)	32 62 (19–81)
“Total interval”*** Time elapsed between first symptoms and treatment initiation	110 73 (77–150)	128 96 (90–186)	124 123 (72–195)	94 100 (54–154)	85 750 (85–835)

*n*, number of cases; IQR - Interquartile range; P25 - 25th percentile; P75 - 75th percentile; * Not including women diagnosed through screening programs (*n* = 164) and women with unknown dates of first symptoms (*n* = 55). ** Not including women diagnosed through screening programs (*n* = 164), referred to specialist care from radiology services (*n* = 236) and women who did not consult primary care services (*n* = 45).

Figure 2 shows a box plot representation of the “TI” in each type of cancer and Table 3 shows the relationship between “TI” duration for each type of cancer and the respective patient-, healthcare- and disease-related variables. In breast cancer, this interval was observed to have a significantly longer duration among women over the age of 50 years having no family history, no participation in screening program and no preferential referral to hospital care. In endometrial cancer, the “TI” was significantly higher among women with family history, those with fewer risk factors, and those whose first consultation took place at a health center. In ovarian cancer, this interval was longer in women with no preferential referral, as well as in those whose first consultation took place at a health center.

**Table 3:** Relationship between duration of “total interval” and patient-, healthcare- and disease-related variables.

Variables	Breast (*n* = 584)		Endometrial (*n* = 52)		Cervical (*n* = 37)		Ovarian (*n* = 43)	
	Total interval Mean (SD)	*p*	Total interval Mean (SD)	*p*	Total interval Mean (SD)	*p*	Total interval Mean (SD)	*p*
Age								
< 50 years	117.3 (70.9)	**0.006**	122.0 (88.4)	0.526	141.2 (88.7)	0.815	109.1 (64.6)	0.831
≥ 50 years	144.0 (118.4)		140.4 (57.5)		152.8 (126.6)		110.5 (74.1)	
Family history in first-degree blood relatives								
Yes	130.4 (91.0)	**0.047**	143.1 (59.0)	**0.028**	150.2 (117.9)	0.804	116.4 (74.0)	0.239
No	139.1 (133.0)		75.0 (13.2)		135.0 (-)		70.8 (37.2)	
Comorbidity								
Yes	127.3 (96.4)	0.422	142.4 (63.2)	0.844	150.3 (91.3)	0.549	80.1 (41.3)	0.089
No	135.6 (104.1)		137.8 (59.3)		148.6 (141.8)		126.5 (79.6)	
Screening program participation								
Yes	125.3 (82.7)	**0.011**	-	-	155.7 (123.6)	0.726	-	-
No	170.0 (151.7)				134.0 (98.4)			
Priority of referral:*								
Standard	212.3 (70.3)	**0.04**	-	-	-	-	204.2 (58.4)	**0.006**
Preferential	129.0 (93.9)		150.2 (57.9)		166.6 (119.9)		115.4 (60.6)	
Place of initial consultation:								
Primary care	130.5 (94.1)	0.740	150.2 (57.9)	**0.002**	166.6 (119.9)	0.081	139.6 (71.2)	**<0.001**
Other departments	114.8 (110.3)		82.4 (28.1)		76.8 (52.6)		67.1 (46.9)	
Stage								
Stage I	124.5 (68.0)	0.309	135.3 (52.7)	0.771	170.7 (111.2)	0.615	118.0 (-)	0.574
Other stages	135.7 (112.8)		145.4 (71.7)		145.9 (119.4)		110.0 (72.6)	
Risk factors								
Fewer than 2	132.3 (105.3)	0.978	171.0 (53.2)	**0.004**	142.8 (77.7)	0.772	100.2 (59.3)	0.681
2 or more	132.0 (96.8)		122.8 (56.7)		151.6 (126.5)		118.8 (81.1)	

*n*, number of cases; <, less than, ≥, greater than or equal to; *p*-value, value of *p*; SD, standard deviation; “-”, insufficient data for these variables. Values in bold indicate statistically significant results. *Not included: women who went directly to hospital.

The most frequent presenting symptoms in cases of breast, endometrial and cervical cancer were breast lump and postmenopausal bleeding, respectively, in both endometrial and cervical cancer. In ovarian cancer, the most frequent symptom was bloating, with the mean “TI” duration being significantly lower (*p* = 0.003) among women who initially presented with this symptom (81.3 days; SD = 55.6) than among those who commenced with other symptoms (148.2 days; SD = 73.8).

Table 4 shows the relationship between the above variables and duration of the “HCI.” In breast cancer, this interval was observed to have a significantly longer duration among women diagnosed in stage I, without preferential referral to hospital care. In endometrial cancer, the “HCI” was significantly higher in women with family history, women with fewer risk factors, and those whose first consultation took place at a health center. In ovarian cancer, this interval was longer among women with no preferential referral, as well as among those whose first consultation took place at a health center. In terms of symptoms of presentation, the “HCI” was significantly lower in women with breast lump (88.8 days; SD = 43.9) versus other symptoms (120.5 days; SD = 64.7), and with bloating (58.0 days; SD = 47.5) versus other symptoms (122.1 days, SD = 81.7), in cases of breast (*p* < 0.001) and ovarian cancer (*p* = 0.01) respectively.

**Table 4:** Relationship between duration of “healthcare interval” and patient-, healthcare- and disease-related variables.

Variables	Breast (*n* = 584)		Endometrial (*n* = 52)		Cervical (*n* = 37)		Ovarian (*n* = 43)	
	Healthcare interval Mean (SD)	*p*	Healthcare interval Mean (SD)	*p*	Healthcare interval Mean (SD)	*p*	Healthcare interval Mean (SD)	*p*
Age								
< 50 years	91.0 (51.5)	0.628	93.5 (56.1)	0.601	111.2 (58.2)	0.459	83.0 (69.7)	0.923
≥ 50 years	94.0 (45.2)		113.9 (48.4)		96.0 (62.8)		86.4 (72.7)	
Family history in first-degree blood relatives								
Yes	92.3 (48.4)	0.710	115.8 (48.1)	**0.013**	99.1 (61.7)	0.509	92.7 (73.8)	0.266
No	94.2 (46.7)		57.7 (6.8)		125.0 (-)		41.0 (23.9)	
Comorbidity								
Yes	92.2 (50.6)	0.732	115.5 (46.9)	0.704	103.0 (53.7)	0.573	55.1 (41.2)	0.088
No	93.0 (46.2)		111.2 (49.8)		97.4 (70.0)		102.3 (78.9)	
Screening program participation								
Yes	95.2 (45.5)	0.555	-	-	106.9 (67.1)	0.613	-	-
No	90.9 (47.5)				83.8 (39.6)			
Priority of referral:*								
Standard	150.7 (38.6)	**0.002**	-	-	-	-	189.2 (63.6)	**0.008**
Preferential	92.0 (47.7)		119.8 (48.3)		107.4 (62.3)		83.2 (61.9)	
Place of initial consultation:								
Primary care	93.0 (48.1)	0.241	119.8 (48.3)	**0.012**	107.4 (62.3)	0.263	112.1 (77.7)	**0.002**
Other departments	54.3 (20.8)		74.4 (31.0)		70.5 (46.8)		47.1 (36.0)	
Stage								
Stage I	99.6 (48.6)	**0.044**	109.1 (36.6)	0.897	132.3 (70.1)	0.269	28.0 (-)	0.174
Other stages	89.4 (47.5)		117.8 (66.4)		95.0 (59.2)		87.3 (71.5)	
Risk factors								
Fewer than 2	91.1 (46.9)	0.367	127.6 (45.4)	**0.041**	120.8 (86.7)	0.620	67.6 (62.7)	0.063
2 or more	94.3 (49.2)		104.5 (49.2)		93.9 (51.8)		101.2 (75.9)	

*n*, number of cases; <, less than, ≥, greater than or equal to; *p*-value, value of *p*; SD, standard deviation; “-”, insufficient data for these variables. Values in bold indicate statistically significant results. *Not included: women who went directly to hospital.

Table 5 shows the relationship between the above variables and duration of “DI.” In breast cancer, this interval was observed to have a significantly longer duration in women under the age of 50 years, with no preferential referral to hospital care. In ovarian cancer, this interval was higher in women with no preferential referral. Moreover, both in breast and ovarian cancers and in endometrial and cervical cancers, “DI” duration was significantly longer in women whose first consultation took place at a health center. In terms of their presenting symptoms of breast cancer, the “DI” was significantly lower (*p* < 0.001) among women with breast lump (45.7 days; SD = 33.4) versus other symptoms (72.8 days; SD = 65.0).

**Table 5:** Relationship between duration of “diagnostic interval” and patient-, healthcare- and disease-related variables.

Variables	Breast (*n* = 584)		Endometrial (*n* = 52)		Cervical (*n* = 37)		Ovarian (*n* = 43)	
	Diagnostic interval Mean (SD)	*p*	Diagnostic interval Mean (SD)	*p*	Diagnostic interval Mean (SD)	*p*	Diagnostic interval Mean (SD)	*p*
Age								
< 50 years	49.5 (44.8)	**0.005**	59.0 (36.0)	0.770	54.1 (42.6)	0.772	66.4 (52.3)	0.791
≥ 50 years	41.0 (28.8)		59.3 (47.1)		52.8 (46.7)		77.7 (69.2)	
Family history in first-degree blood relatives								
Yes	44.14 (35.5)	0.901	60.9 (46.6)	0.239	53.5 (45.5)	0.824	78.7 (67.6)	0.363
No	44.6 (37.9)		33.3 (31.3)		52.0 (33.6)		45.4 (24.8)	
Comorbidity								
Yes	47.06 (39.8)	0.142	65.6 (46.9)	0.849	49.8 (41.9)	0.428	53.3 (36.0)	0.179
No	42.4 (33.1)		58.4 (46.4)		57.7 (47.7)		88.9 (75.6)	
Screening program participation								
Yes	40.9 (31.7)	0.205	-	-	55.1 (47.2)	0.846	-	-
No	45.6 (34.2)				46.1 (29.7)			
Priority of referral:*								
Standard	121.5 (47.0)	**0.000**	-	-	-	-	190.7 (67.5)	**0.002**
Preferential	48.2 (38.9)		68.1 (46.7)		54.1 (56.9)		76.3 (45.8)	
Place of initial consultation								
Primary care	49.5 (39.9)	**0.040**	68.1 (46.7)	**0.001**	64.1 (56.9)	**0.025**	104.9 (71.4)	**<0.001**
Other departments	26.5 (13.8)		22.3 (13.9)		22.3 (17.2)		36.8 (23.8)	
Stage								
Stage I	44.5 (38.0)	0.893	55.6 (38.4)	0.840	58.8 (42.4)	0.177	35.0 (-)	0.600
Other stages	44.1 (34.8)		66.3 (58.5)		49.2 (46.2)		75.8 (65.3)	
Risk factors								
Fewer than 2	44.4 (37.4)	0.902	67.5 (48.6)	0.287	54.2 (61.5)	0.421	64.6 (59.8)	0.365
2 or more	44.0 (34.5)		55.3 (44.9)		53.1 (37.4)		82.9 (68.7)	

*n*, number of cases; <, less than; ≥, greater than or equal to; *p*-value, value of *p*; SD, standard deviation; “-”, insufficient data for these variables. Values in bold indicate statistically significant results. *Not included: women who went directly to hospital.

The “DI” was significantly lower (*p* < 0.001) in women with breast cancer who had been diagnosed by a screening program (30.3 days; SD = 14.8) than among the rest (49.1 days; SD = 39.7), without there being any differences in the “TTI.” In the case of cervical cancer, no statistically significant differences in “DI” were observed among women diagnosed by a screening program, yet their “TTI” was significantly longer (*p* = 0.01) (85.7 days; SD = 51.1) than that of those who had been diagnosed after presenting with symptoms (45.8 days; SD = 27.4).

Only 6 women had presented with vulvar cancer, with all being over 50 years of age and four being in stage I. Four of them had attended their first consultation at a health center and been preferentially referred to hospital care. The symptom of presentation in 5 of these women was vulvar ulcer.

A logistic regression model, which included the variables that had shown a statistically significant association, showed that the variables associated with a “TI” of more than 90 days in cases of breast cancer were age 50 years or over (OR = 1.8; CI 95% = 1.1–2.7), and symptoms of presentation other than breast lump (OR = 4.6; CI 95% = 1.8–12.0) (Table 6). In the case of any given gynecological cancer, the associated variables were absence of family history of gynecological cancer in first-degree blood relatives (OR = 7.2; CI 95% = 1.3–39.1), and having attended a health center as the place of first consultation (OR = 5.3; CI 95% = 2.0–13.8).

**Table 6:** Variables related to a “TI” > 90 days in breast and gynecological cancer according to logistic regression.

Type of cancer	Variables	Regression coefficient	Wald	*p*	OR (95% CI)
Breast*	Age (≥ 50 years)	0.6	6.5	0.011	1.8 (1.1–2.7)
	Symptoms other than a lump in the breast	1.5	9.7	0.002	4.6 (1.8–12.0)
Gynecological**	First-degree family history of gynecological cancer	2.0	5.3	0.022	7.2 (1.3–39.1)
	First consultation at the health center	1.7	11.6	<0.001	5.3 (2.0–13.8)

*Number of events: 393; Independent variables included in the model: 5; Area Under the Curve: 0.621. **Number of events: 68; Independent variables included in the model: 4; Area Under the Curve: 0.694; ≥ − greater than or equal to; *p*-value, value of *p*; OR, Odds ratio. CI, Confidence interval; %, percentage.

Logistic regression likewise showed that the variables associated with a “DI” of more than 30 days in breast cancer were presence of fewer than 2 risk factors (OR = 1.6; CI 95% = 1.1–2.3), and in the case of any given gynecological cancer, the fact of having attended the first consultation at a health center (OR = 5.5; CI 95% = 2.4–12.6) (Table 7).

**Table 7:** Variables related to a “DI” > 30 days in women with breast and gynecological cancer according to logistic regression.

Type of cancer	Variables	Regression coefficient	Wald	*p*	OR (95% CI)
Breast*	Fewer than 2 risk factors	0.455	5.268	0.022	1.6 (1.1–2.3)
Gynecological**	First consultation at the health center	1.705	16.173	<0.001	5.5 (2.4–12.6)

*Number of events: 329; Independent variables included in the model: 4; Area Under the Curve: 0.583; **Number of events: 92; Independent variables included in the model: 2; Area Under the Curve: 0.681. ≥ − greater than or equal to; *p*-value, value of *p*; OR, Odds ratio; CI, Confidence interval; %, percentage.

## Discussion

In the study sample, which was predominantly made up of women with breast cancer, the majority were over 50 years of age, and presented with comorbidity and risk factors for each type of cancer but no family history of the same cancer. Most tumors of the breast and cervix had not been diagnosed by screening programs. Most patients had been diagnosed in the early stages, except for those with ovarian tumors, which were largely diagnosed at advanced stages. Insofar as the most frequent symptoms of presentation were concerned, these were breast lump for breast cancer, postmenopausal bleeding for both endometrial and cervical cancer, bloating in the case of ovarian cancer, and vulvar ulcer in the case of vulvar cancer. Excluding those patients who had been diagnosed by screening programs, the most frequent place of consultation of first symptoms for all types of cancer was the GP’s practice.

Primary care is the primary setting where cancer is first suspected, and most people with cancer present with symptoms that prompt consultation with their family doctor (16). Most people with cancer continue to be diagnosed after they have symptoms (17), even though population screening is available for some of these conditions. The reason why more breast and cervical cancers are diagnosed through symptoms than through screening programs is undoubtedly multifactorial and related to clinical, organizational and population factors. On the one hand, screening tests have variable sensitivity and also false negatives. In addition, coverage and adherence to screening must be taken into account, with geographical, cultural or personal barriers coming into play. On the other hand, it should be noted that some cancers are more aggressive and grow rapidly between two screening tests, which contributes to detection through symptoms (4).

The results, showed that the longest “PI” was registered for vulvar cancer, and though far higher than the “PCI,” whose longest duration corresponded to ovarian cancer, it was nevertheless lower than the “HCI,” which exceeded 80 days in all types of cancer. The mean “PI” duration was 20 to 50 days, except in vulvar cancer where it reached 248 days. In ovarian cancer, with a “PI” of around 25 days, the duration was very similar to that reported in a recent study covering a number of countries, in which this period ranged from 21 to 35 days (18). In breast cancer, the results obtained for the “PI,” i.e., approximately 36 days, were lower than those published by Petrova et al. in a systematic review of 50 studies conducted in different countries, in which the mean duration was 50 days (14).

Our results showed a higher “DI” for breast and ovarian cancer than that reported by two studies undertaken in the USA (19, 20). In cervical and endometrial cancer, however, our “DI” proved to be lower than that reported in studies undertaken in the Netherlands (21) and Brazil (22) respectively. The median “DI” figure ranged from 35 to 55 days, with ovarian neoplasms registering the longest and breast neoplasms the shortest durations, respectively. A greater delay in the “DI” was observed in the case of breast cancer among women with fewer than 2 risk factors, and in the case of gynecological cancer among women whose first consultation took place at a health center.

The greatest delay in the “DI” of ovarian cancer may be related to the appearance of vague initial symptoms that are easily attributed to other common and more trivial conditions. In addition, there is a lack of awareness about this disease, with a low index of suspicion, which contributes to delays in referral and diagnosis (23).

GPs tend to be patients’ first contact with the health service, and it falls to them to decide which tests must be performed, and when and where to refer the patient (21). The main challenge for the family doctor is to maintain a difficult balance between avoiding unnecessary interventions and not delaying necessary actions in the face of alarm symptoms (24). According to a study conducted in the United Kingdom, 80% of patients diagnosed with cancer had previously consulted their GPs once or twice before being referred to secondary care, and the remaining 20% required three or more consultations (25). In our results, the “PCI,” which can influence the prognosis of the disease, registered a median of 0 to 18 days, a figure very similar to that described by Vedsted et al. (26) in different countries, though higher than the interval of 7 days reported by Koo et al. (12), in a study conducted in England.

In certain healthcare systems, GPs suggest that patients participate in opportunistic screening tests to increase population participation in screening programs. In the study conducted by Luo (27) to determine the effectiveness of different screening programs worldwide, he observed that countries where opportunistic screening was carried out, such as the USA, had lower participation rates than countries where screening was carried out on a widespread basis, such as the United Kingdom (51.3% vs. 83.6%).

A previous study (28) found that for every 4 weeks of delay in treatment initiation, mortality increases significantly in different types of cancer, such as those of the breast and cervix. In our results, save for ovarian cancer, the “TTI” exceeded the 28 days proposed by Hanna et al. (28) The time interval between diagnosis and treatment initiation influences the prognosis of the disease, determining an increase in mortality (29, 30). While primary prevention will doubtless lead to a reduction in the incidence of most tumors in the long term, in the short term a reduction in cancer-related mortality calls for improvements, not only in early detection in primary care, but also in diagnosis and treatment at the specialized level (31, 32).

Our study observed that the median “HCI” ranged from 54 to 106 days, a period which is higher in all cancers (excluding ovarian cancer) than the median estimated by Hansen et al. (33) in Denmark. The factors that account for the differences found between countries include, among others, access to different complementary tests, the number of patients allocated to each GP, and waiting lists in hospital care. The “HCI” for all cancers, except endometrial cancer, constituted the longest time interval, with the components that play a leading role being “DI” and “TTI,” and to a lesser extent, “PCI.”

With respect to the “TI,” the median ranged from 85 to 128 days, with this being highest in endometrial cancer and lowest in vulvar cancer. In all cancers, the mean value of this interval was 133 days, a figure higher than that described by Vandborg et al. (34) in a study targeted at ascertaining the relationship between delay on the one hand, and the characteristics of patients and the health system on the other. In our case, we found evidence of a longer delay in breast cancer among women over 50 years of age, and those with presenting symptoms other than breast lump. In gynecological cancers, the variables associated with a longer delay were absence of family history of gynecological cancer in first-degree blood relatives and having attended the health center as the place of initial consultation. A study conducted by Ramírez (35) ascertained that, in general, patients with a “TI” of more than 90 days registered worse results in terms of survival. Hence, measures capable of reducing this total interval from symptom onset to treatment initiation should be implemented.

The results of the study have made it possible to quantify different time intervals in the diagnosis of breast and gynecological cancer and to identify the variables that influence the duration of these intervals. The results provide useful information for proposing improvements in accessibility to diagnostic and therapeutic resources in our environment, as well as for implementing preferential referral circuits that contribute to improving early detection and, consequently, the prognosis of the disease (36). Some countries have introduced a pathway for cancer patients, often called “the fast track,” aimed at shortening the time between consultation and treatment in cases of suspected cancer (37). Good results have been achieved in several countries, with improved diagnostic times and improved survival rates.

The GPs are directly involved in the initial diagnosis of most cancer cases. Several factors can play a role in raising awareness of the disease, such as appropriate clinical knowledge of warning signs and the epidemiology of each tumor, but also knowing patients well and being alert to changes in their appearance or behavior. A Danish cohort study found that there was a greater delay in situations where the physician had high care pressure and little prior knowledge of his patients (38). In all cases, the family doctor’s response to suspected cancer should be to make an appropriate diagnosis in collaboration with the hospital, using the necessary procedures (39).

In conclusion, most patients with breast and/or gynecological cancer are diagnosed in early stages of the disease, except in the case of ovarian cancer where diagnosis at advanced stages is more frequent. The majority of breast and cervical tumors are not diagnosed by screening programs. The time interval that features most prominently in the Total interval is the Diagnostic interval from first consultation until diagnosis, which includes the Primary care interval. The Treatment interval from diagnosis until treatment initiation is high for the majority of tumors, and is longer than the recommended time. In breast cancer, the Total interval from symptom onset to treatment initiation, is longer in women over the age of 50 years and in those with presenting symptoms other than breast lump. With respect to gynecological cancers, this interval is higher in cases where there is no family history, and where the health center is the first place of consultation. The results provide useful information for proposing improvements in access to diagnostic and therapeutic resources, and also for implementing preferential referral circuits that contribute to improving early detection and prognosis of the disease.

As for the limitations of the study, we observed some variability in the degree of completion of the medical records, with information on some of the variables studied sometimes being deficient. To avoid bias in the measurement of time intervals in cancer diagnosis, we followed the recommendations contained in the Aarhus Declaration, which includes a useful checklist for the design of this type of study and adequately defines the time points that delimit the time intervals. It is necessary to point out the low statistical power in cases of endometrial, cervical, ovarian, and vulvar cancer due to the small number of cases, especially in the latter tumor, in which there were only six women, which explains the wide confidence intervals in some estimates. It is possible that potential confounding factors may have been omitted in the selection of study variables, and the generalization of the results to other areas of health may be limited.

## Data Availability

The raw data supporting the conclusions of this article will be made available by the authors, without undue reservation.

## Glossary

“-”: Insufficient data for these variables
>: Greater than
<: Less than
%: Percentage
CI: Confidence interval
DI: Diagnostic Interval
GPs: General practitioners
HCI: Healthcare interval
HOSPI: Hospital care interval
ICD-9: International Classification of Diseases, 9th Revision
ICPC-2: International Classification of Primary Care, 2nd edition
IQR: Interquartile range
*n*: Number of cases
NICE Guidelines: National Institute for Health and Care Excellence
OR: Odds ratio
*p*-value: Value of *p*
P25: 25th percentile
P75: 75th percentile
PCI: Primary care interval
PHI: Physician interval
PI: Patient interval
PPV: Positive predictive value
SD: Standard deviation
TI: Total interval
TTI: Treatment interval
